# Burden of adult neurofibromatosis 1 questionnaire: translation and psychometric properties of the Persian version

**DOI:** 10.1186/s13023-023-02681-x

**Published:** 2023-06-23

**Authors:** Reza Jahanshahi, Zahra Yasaghi, Fatemeh Mirzaei, Shohreh Ghasemi, Akram Sanagoo, Leila Jouybari, Samira Foji

**Affiliations:** 1 BSN, Student Research Committee, Golestan University of Medical Sciences, Gorgan, Iran; 2 Master of critical care nursing, school of nursing and midwifery, Golestan University of Medical Sciences, Gorgan, Iran; 3 DDS, Student Research Committee, Golestan University of Medical Sciences, Gorgan, Iran; 4 DDS, MSc of Oral Surgery, Adjunct Clinical, Department of Augusta University, GA Augusta, USA; 5 School of nursing and midwifery, Golestan University of Medical Sciences, Gorgan, Iran; 6 School of nursing and midwifery, Golestan University of Medical Sciences, Gorgan, Iran; 7 School of Nursing and Midwifery, Sabzevar university of medical scinces, Sabzevar, Iran

**Keywords:** Cost of illness, Iran, Psychometrics, Rare diseases, Von Recklinghausen’s disease

## Abstract

**Background:**

The notion of “burden” has taken a key place in the evaluation of care, particularly in the case of rare diseases. The aim of this study was to evaluate the psychometric properties of the burden of neurofibromatosis 1 questionnaire (BoN) and to determine the perceived disease burden.

**Results:**

The 15-item BoN was translated into Persian, and no items were removed based on content validity. The adequacy of the sample was acceptable (KMO = 0.902), and Bartlett’s test of sphericity revealed statistically significant results (P < 0.001). Exploratory factor analysis revealed three factors. The reliability of the scale was good (Cronbach’s alpha: 0.90), and the intraclass coefficient was 0.85. The severity of the burden of neurofibromatosis was moderate, and the total mean burden score was 33.12 ± 16.12.

**Conclusions:**

The Persian version of the BoN is an acceptable tool in terms of structure and content, and it specifically assesses the practical aspects of daily activities for patients with neurofibromatosis.

## Introduction

Neurofibromatosis Type 1 (NF1), or Von Recklinghausen’s disease, is the most common autosomal dominant neuroectodermal disease; it is primarily characterized by the presence of six or more café-au-lait macules, intertriginous freckles, and two or more neurofibromas [1–3]. NF1 is the most common type of this disease, with an estimated prevalence of approximately 1:2000–1:3500 individuals around the world [4, 5]. Signs and symptoms reduce the quality of life significantly; they are not life-threatening, but they can cause severe morbidity [6].

NF1 is usually diagnosed clinically by dermatologists or pediatricians since it affects the skin at an early age and the nervous system at a later age. Genetic tests could not be performed routinely in low-income countries. The chief complaints of NF1 patients are mostly cosmetic deformities, chronic pain and difficulty learning, which subsequently lead to social complications, low self-esteem, depression and mental disorders. Previous studies have shown that the prevalence of depression and other psychiatric complications was higher in patients with NF1 than in healthy adults [7, 8].

There is no definitive treatment for NF1, but in cases of malignancies, treatment options include surgery, chemotherapy and radiotherapy. For the proper palliative management of NF1 patients with numerous psychiatric and psychological disorders, a systematic multidisciplinary approach must be considered, including coordination among dermatologists, neurologists, surgeons, psychologists and other health care workers involved with such patients. Health care professionals should also be familiar with health-related concepts such as “individual disease burden”, which assesses disease “disability” in the broadest sense, including social, psychological, physical, and economic features. The term “individual burden” derives from the general concept entitled “Global Disease Burden”, which was introduced in 2010 by the World Health Organization (WHO), for quantifying population health and determining action priorities [9]. Individual burden has been increasingly investigated, and different studies have created and validated specific assessment tools for the burden of each skin disease, such as psoriasis, vitiligo, atopic dermatitis, hereditary ichthyosis, and infantile hemangioma [10–14].

The individual disease burden of neurofibromatosis was investigated first by Armand et al. in 2019 [15]. They developed and validated a questionnaire in the French language entitled “Burden of Neurofibromatosis” (BoN) and investigated it among 60 adult patients. As the second step, they translated the tool from French to English. The scale exhibited strong psychometric properties, including high internal consistency (α = 0.91). To the best of our knowledge, there is no Persian-language tool to measure the concept of burden, so we aimed to translate, culturally adapt and validate the BoN and investigate the psychometric properties of the Persian version of the BoN.

## Method

The methodological study was conducted as follows: (a) translation and adaptation of the BoN to Persian in 7 steps [16]; (b) assessment of the psychometric properties of the questionnaire.

## Measures

The following demographic information was collected using a self-report questionnaire: age, gender, level of education, marital status, and employment status.

The BoN consists of 15 items with four dimensions (concentration and learning problems (5 items); the way others look at them and the anxiety they feel about the future (5 items); life with the disease (3 items); sexuality (2 items)) and is rated on a 6-point scale, ranging from 0 (never) to 5 (constantly). The total score is calculated by summing the scores of all items and ranges from 0 to 75. Higher scores indicate higher levels of neurofibromatosis burden [15].

## Participants

Four hundred patients with neurofibromatosis were invited to participate in this study. The inclusion criteria for participants were as follows: (a) over 18 years old; (b) neurofibromatosis disease as defined by the Iranian Neurofibromatosis Association (physicians consider the following to diagnose the NF1: Family history review, genetic testing, café-au-lait macule size review, etc. [17]); and (c) no history of psychiatric illness. The questionnaire was administered electronically via the Google Forms platform, the hyperlink was sent to the participants. A total of 356 people completed the questionnaire. Data collection took place between January 2020 and February 2020.

## Translation process

Initially, two bilingual translators independently translated the questionnaire from English to Persian. Subsequently, a third bilingual translator compared the two translations and combined them into a single version. The text of the current questionnaire was then translated back into the original language by another bilingual translator and compared to the original version of the questionnaire. Following the translation, the questionnaire’s items were culturally verified; in the end, no cultural alterations were made because the questions were clear and intelligible to Iranian society. The text of the questionnaire was shown to 10 patients with neurofibromatosis. We asked them to review the sentences for clarity, comprehensibility, simplicity and to comment on how the items could be improved. In accordance with suggestions from the patients, we replaced the word “neurofibromatosis” with the word “disease” because they reported feeling bad when reading the former word. After applying the final corrections, the final version was prepared, and its psychometric properties were assessed.

## Psychometric Procedure

Content validity is assessed using the Content Validity Ratio (CVR) and Content Validity Index (CVI). Twenty experts reviewed the questionnaire items and rated each one as essential, useful but not necessary, or unnecessary [18]. According to the Lawshe table, the acceptable rate is 0.42 [19]. None of the phrases were deleted in this section. After applying expert opinions and enriching the phrases, 15 other experts reviewed the questionnaire to evaluate the CVI [20]. The construct validity was assessed by exploratory factor analysis (EFA) and confirmatory factor analysis (CFA). Additionally, 178 participants were used for each method.

### Statistical analysis

To analyze the psychometric properties of the BoN, Lisrel version 8.80 and SPSS version 18.0 were used. The basic construct of the items was assessed by EFA using principal component analysis with direct oblimin rotation. Bartlett’s test of sphericity (BTS) was used to assay the correlation matrix between items (p < 0.05). The Kaiser–Meyer–Olkin index (KMO) test was used to assess the quality of sampling [21], and the Kaiser index was used to estimate the number of factors. Confirmatory factor analysis was performed to evaluate the fit of the model. The goodness-of-fit index (GFI), normed fit index (NFI), nonnormed fit index (NNFI), comparative fit index (CFI), root mean square error (RMSEA), and degrees of freedom (CMIN/DF) were used, as they are commonly used fit indices for CFA [22]. Cronbach’s alpha coefficient was calculated to assess the internal consistency [23]. A test-retest analysis was conducted to assess replicability by asking a group of subjects to complete the questionnaire twice, with an interval of at least 14 days in between [24].

## Results

Three hundred fifty-six patients participated in the present study. Demographic information from the participants is shown in Table 1. Most of the participants were female and single. The average age of the participants was 34.22 ± 8.6 years (Table 2).

**Table 1:** Demographic characteristics and responses on the burden of adult neurofibromatosis 1 questionnaire

Variable		EFA sample (n = 178)	CFA sample (n = 178)	Total (n = 356)	BoN	*P*-value
		n (%)	n (%)	n (%)	Mean (± SD)	
Age group						
	18–25 yrs	20 (11.2)	36 (20.2)	56 (15.7)	37.26 (± 18.00)	0.04
	26–35 yrs	74 (41.6)	77 (43.3)	151 (42.4)	33.98 (± 16.62)	
	36–45 yrs	68 (38.2)	52 (29.2)	120 (33.7)	31.16 (± 15.28)	
	> 46 yrs	16 (9.0)	13 (7.3)	29 (8.1)	28.68 (± 15.01)	
Gender						
	Female	131 (73.6)	115 (64.6)	246 (69.1)	32.75 (± 15.51)	0.55
	Male	47 (26.4)	63 (35.4)	110 (30.9)	33.93 (± 18.27)	
Marriage						
	Single	122 (68.5)	120 (67.4)	242 (68.0)	34.74 (± 17.23)	0.003
	Married	56 (31.5)	58 (32.6)	114 (32.0)	29.66 (± 13.90)	
Education						
	Diploma and sub-Diploma	97 (54.5)	97 (54.5)	194 (54.5)	35.63 (± 17.08)	0.001
	Bachelor’s degree	57 (32.0)	62 (34.8)	119 (33.4)	32.00 (± 15.10)	
	Master’s degree	24 (13.5)	17 (9.6)	41 (11.5)	25.34 (± 13.83)	
	Doctoral degree	0 (0)	2 (1.1)	2 (0.6)	15.00 (± 4.24)	
Job						
	Unemployed	52 (29.2)	55 (30.9)	107 (30.1)	41.20 (± 15.39)	0.001
	Self-employment	48 (27.0)	44 (24.7)	92 (25.8)	28.55 (± 17.37)	
	Housewife	39 (21.9)	44 (24.7)	83 (23.3)	32.46 (± 15.17)	
	Employee	39 (21.9)	35 (19.7)	74 (20.8)	29.28 (± 14.61)	

Values are presented as n (%), Mean and Standard deviation (± SD)

Significant at *p* < 0.05.

**Table 2:** KMO and Bartlett’s test

Kaiser-Meyer-Olkin Measure of Sampling Adequacy		0.902
Bartlett’s Test of Sphericilty	Approx. Chi-Square	1272.551
	df	105
	Sig.	< 0.001

In the process of evaluating the content validity, no items were removed from the questionnaire, but changes were made in the form and richness of the words to enhance understanding. The CVR value ranged from 0.5 to 0.9 for the individual items and 0.74 for the whole scale. The adequacy of the samples shown by the results of the EFA (KMO = 0.902) (Table 1). The BTS results were statistically significant (P < 0.001) and led to the development of a three-factor solution as a domain. The CFA confirmed the following three factors:

Factor 1, concentration and life with the disease (six items);

Factor 2, the social burden of illness and future worries (six items);

Factor 3, perspectives (three items).

The factor loadings of the 3-factor solution are shown in Table 3. The resulting domains accounted for 61.012% of the observed variance in the 15-item BoN. The fit indices for the BoN are shown in Table 4.

**Table 3:** Factor loadings of the BoN on the rotated factor pattern matrix

No	Factor 1	Factor 2	Factor 3
q1	0.746		
q2	0.897		
q3	0.776		
q4	0.700		
q5	0.506		
q6			0.427
q7		0.856	
q8		0.894	
q9		0.398	
q10		0.513	
q11	0.498		
q12			0.534
q13			0.825
q14		0.749	
q15		0.548	

**Table 4:** Model Fit Index Summary

Model Fit Index	Adminissibility	Result
χ^2^*P*-value (Chi-squared *P*-value)	> 0.05	> 0.001
RMSEA (Root Mean Square Error of Approximation)	< 0.08 perfect fit; 0.08-0.10 good fit; >0.10 weak fit	0.094
NFI (Normed Fit Index)	> 0.9	0.94
NNFI (Non Normed Fit Index)	> 0.9	0.96
GFI (Goodness of Fit Index)	> 0.9	0.91
CFI (Comparative of Fit Index)	> 0.9	0.97
CMIN/DF (Minimum Discrepancy Function by Degree of Freedom divided)	< 3 good; <5 sometimes permissible	2.45

The Cronbach alpha coefficient of the total scale was equal to 0.90, and the coefficients for factors 1, 2, and 3 were 0.85, 0.85, and 0.53, respectively. The intraclass correlation coefficient for the total scale was 0.85. The test-retest reliability was assessed by examining results from 30 subjects, and the questionnaire was found to have good replicability.

## The burden of adult neurofibromatosis 1 questionnaire

The participants’ scores on the burden of neurofibromatosis 1 questionnaire is reported in Table 5. The total mean score of burden was 33.12 ± 16.12, with a range between 0 and 75. The mean scores for the three questionnaire factors “concentration and life with the disease”, “the social burden of illness and future worries”, and “perspectives” were 10.36 ± 6.97, 17.23 ± 7.98 and 5.52 ± 3.71, respectively. Table 2 shows the relationship between the burden of neurofibromatosis 1 scores and demographic variables.

**Table 5:** participants’ Burden of neurofibromatosis 1 by factors (N = 356)

		Never,%	Rarely,%	Sometimes, %	Often, %	very often, %	Constantly, %
concentration and life with the disease	Do you think that your concentration problems have had a negative impact on your work?						
		11	23	40.2	3.9	14.9	7
	Do you think that your concentration problems have restricted your daily activities?						
		20.2	27.5	24.4	12.1	9.8	5.9
	During your education, do you think that you had learning difficulties because of your NF1?						
		24.2	20.5	19.1	9.3	16.6	10.4
	Do you think that your concentration problems have hindered your inclusion in society?						
		43.8	20.5	20.2	3.4	7.3	4.8
	Have you had any difficulties in asking for help?						
		32.6	23	23.6	6.2	8.4	6.2
	Has the paperwork in connection with your NF1 been difficult?						
		32	24.2	20.2	7.3	5.1	11.2
social burden of illness and future worries	Because of your NF1, has the way other people look at you caused you to suffer?						
		8.1	14.6	27.5	5.6	17.1	27
	Has your NF1 affected which clothes you choose to wear?						
		10.7	9.8	10.4	3.9	20.5	44.7
	Have you felt that you have no control over what is happening to you?						
		17.7	22.2	30.6	5.3	10.7	13.5
	Are you sometimes afraid of the future because of your NF1?						
		6.7	6.7	18	7	18.5	43
	Do you think your NF1 has made you shyer?						
		9.8	14	18	7.9	17.1	33.1
	Has your NF1 hindered your sexuality?						
		31.7	13.8	16.6	3.7	13.8	20.5
Perspectives	Have you felt that your socio-economic status may be directly linked to your NF1?						
		37.4	15.4	18.5	7.3	8.7	12.6
	Have you felt the need to justify yourself?						
		25.6	18	16.9	5.1	16.3	18.3
	Have you perceived your NF1 as a physical disability?						
		31.7	23.9	19.4	11.5	8.1	5.3

## Discussion

The use of valid questionnaire to assess the burden of patient diseases has become an important task in community management [25], and one of the challenges mentioned in a previous review study is the heterogeneity of measurement tools used to assess disease burden [26]. The current study examines the Persian version of the burden of neurofibromatosis type 1 questionnaire; assesses the validity and reliability of the questionnaire; and determine the perceived burden of patients with NF 1. The Persian version of the BoN has good validity and reliability and can be used as a reliable tool for adult patients with NF 1.

The present study was performed with 356 participants. Based on the KMO test, the sample size is excellent and adequate. The KMO value varies from zero to one, 0.5 to 0.6 sample size is not enough and 0.9 to 1 is reported as excellent. [21]. The biggest limitation mentioned by Armand et al. was a relatively small sample size (65 patients) [15]. The KMO test in their study was 0.6. We were able to overcome this limitation.

In this study, we used CFA to examine the validity of the internal structure of scales [27]. All fit indicators were within an acceptable threshold, indicating confidence in the validity of the internal structure. Armand et al. also mentioned the acceptability of fit indices in their study [15].

The reliability of the BoN was 0.9, and the three determined factors also had acceptable values. Its acceptable value is estimated based on studies > 0.70 [23]. The original version reported that the instrument reliability for the whole scale was 0.91 [15].

In this study, the severity of the burden of neurofibromatosis was moderate. Age, marital status, occupation and education affected the patient’s perceived burden. With increasing age, the perceived burden of the disease by patients’ decreases significantly, this result was not far from the mind because this disease seriously affects the appearance of patients and young people care more about their appearance than older people. Patients who are single significantly perceived more burden of disease, but increasing their education degree and having a source of income help to reduce the burden of disease. Policymakers should note that social and economic support can significantly help reduce the perceived burden of disease. Armand et al. reported a moderate severity of disease burden and a relationship between the BoN and sex [15]. Foji et al. identified 4 main categories for the burden of neurofibromatosis type 1 in a qualitative study in Iran, including “deprivation and restriction”, “social isolation”, “ineffective adaptation to the disease”, and “failing and falling behind in life”, which indicates the perception of patients with NF [8]. Kenborg et al. showed in their study that people with NF1 have recurrent clinical problems that persist and accumulate throughout life. Quantification improves our understanding of the conceptual complexities of disease burden [28]. It is suggested that public awareness regarding this disease should be put on the agenda, this action is the basis for solving many social and economic problems of NF patients [29].

One of the strengths of the present study is its sample size, despite the rarity of this disease and the small sample sizes in most studies and the collection of samples from across the country. On the other hand, as we are translating and BoN psychometrics for the first time and no questionnaire in Iran specifically addressed this issue, it was very difficult to compare the situation. We suggest that researchers identify future factors affecting perceived disease burden. Limitations of the study include the use of convenience sampling rather than random sampling. In addition, only those who could read, write, and access the internet were able to participate in the study. Therefore, the burden of participants cannot be generalized to the whole community. In addition, we did not assess the clinical features of the research subjects. We propose that future research quantify clinical features and assess their influence on perceived disease burden.

## Conclusion

The Persian version of the BoN is an acceptable tool in terms of structure and content that specifically addresses the practical aspects of daily activities for patients with neurofibromatosis, beyond the concept of quality of life. NF1 is a disease with wide dimensions, and this tool can be used to better understand the individual burden of patients and play a role in decision-making.

## Data Availability

All data generated and analyzed during this study are available in the published manuscript.
